# Pregnant women’s views on how to promote the use of a decision aid for Down syndrome prenatal screening: a theory-informed qualitative study

**DOI:** 10.1186/s12913-018-3244-1

**Published:** 2018-06-08

**Authors:** Titilayo Tatiana Agbadjé, Matthew Menear, Michèle Dugas, Marie-Pierre Gagnon, Samira Abbasgholizadeh Rahimi, Hubert Robitaille, Anik M. C. Giguère, François Rousseau, Brenda J. Wilson, France Légaré

**Affiliations:** 1 Canada Research Chair in Shared Decision Making and Knowledge Translation, Quebec, Canada; 2 Université Laval Primary Care Research Centre (CERSSPL-UL), Quebec, Canada; 3 Department of Family Medicine and Emergency Medicine, Faculty of Medicine, Université Laval, Quebec, Canada; 4 Faculty of Nursing, Université Laval, Quebec, Canada; 5 Quebec Centre of Excellence on Aging, Quebec, Canada; 6 Department of Molecular Biology, Medical Biochemistry and Pathology, Faculty of Medicine, Université Laval, Quebec, Canada; 7 MSSS/FRQS/CHUQ Research Chair in Health Technology Assessment and Evidence Based Laboratory Medicine, CHU de Québec, Quebec, Canada; 8 School of Epidemiology and Public Health, University of Ottawa, Ottawa, Canada; 9 Centre intégré universitaire de santé et services sociaux (CIUSSS) de la Capitale-Nationale, Pavillon Landry-Poulin, entrée A-1-2, bureau A-4574, 2525, Chemin de la Canardière, Quebec, QC G1J 0A4 Canada

**Keywords:** Behaviour change wheel, Behaviour change techniques, Theoretical domains framework, Theory of planned behaviour, Patient decision aid, Intervention, Promotion, Down syndrome prenatal screening, Pregnant women, Shared decision making

## Abstract

**Background:**

For pregnant women and their partners, the decision to undergo Down syndrome prenatal screening is difficult. Patient decision aids (PtDA) can help them make an informed decision. We aimed to identify behaviour change techniques (BCTs) that would be useful in an intervention to promote the use of a PtDA for Down syndrome prenatal screening, and to identify which of these BCTs pregnant women found relevant and acceptable.

**Methods:**

Using the Behaviour Change Wheel and the Theoretical Domains Framework, we conducted a qualitative descriptive study. First, a group of experts from diverse professions, disciplines and backgrounds (eg. medicine, engineering, implementation science, community and public health, shared decision making) identified relevant BCTs. Then we recruited pregnant women consulting for prenatal care in three clinical sites: a family medicine group, a birthing centre (midwives) and a hospital obstetrics department in Quebec City, Canada. To be eligible, participants had to be at least 18 years old, having recently given birth or at least 16 weeks pregnant with a low-risk pregnancy, and have already decided about prenatal screening. We conducted three focus groups and asked questions about the relevance and acceptability of the BCTs. We analysed verbatim transcripts and reduced the BCTs to those the women found most relevant and acceptable.

**Results:**

Our group of experts identified 25 relevant BCTs relating to information, support, consequences, others’ approval, learning, reward, environmental change and mode of delivery. Fifteen women participated in the study with a mean age of 27 years. Of these, 67% (*n* = 10) were pregnant for the first time, 20% (*n* = 3) had difficulty making the decision to take the test, and 73% had made the decision with their partner. Of the 25 BCTs identified using the Behaviour Change Wheel, the women found the following 10 to be most acceptable and relevant: goal setting (behaviour), goal setting (results), problem solving, action plan, social support (general), social support (practical), restructuring the physical environment, prompts/cues, credible sources and modelling or demonstration of the behaviour.

**Conclusions:**

An intervention to promote PtDA use among pregnant women for Down syndrome prenatal screening should incorporate the 10 BCTs identified.

## Background

In most industrialised countries, screening for Down syndrome (DS) early in the pregnancy is a routine part of prenatal care [1] and is typically offered to all women regardless of their age. DS prenatal screening indicates the probability of the baby having DS [2]. However, DS prenatal screening can result in false positives that produce needless anxiety, or lead to unnecessary diagnostic testing with the risk of miscarriage [1, 3]. Positive results also lead to increasingly difficult decisions, such as whether to do a more risky and invasive test (amniocentesis) and, possibly, whether to terminate the pregnancy or not [2, 4]. Many women are ambivalent about DS screening and diagnosis, yet they are not always informed about its potential benefits and risks or actively involved in the decision-making process [1, 5]. About 6% of pregnant women experience clinically significant decisional conflict regarding prenatal screening for DS that may lead to regret and litigation [6]. Given the difficult and value-laden nature of this decision [7], effective decision support is needed [8].

A patient decision aid (PtDAs) is an evidence-based knowledge tool that provides information on a condition, options for treatment, and probabilities and scientific uncertainties regarding associated benefits and harms [9, 10]. PtDAs help patients participate more actively in decisions and make choices more consistent with their values and preferences [11, 12]. In the context of genetic testing, pregnant women experience lower decisional conflict using a PtDA [13]. Despite these favourable outcomes, their overall use in routine clinical practice is limited [14, 15], including in the context of prenatal care and DS screening [5, 6]. But even if PtDAs were routinely available for decisions about DS screening, it seems not all women would use them.

In a study by our team (referred to hereinafter as the Delanoë study), 31% of pregnant women had little intention to use PtDAs for DS prenatal screening (i.e. their intention was “weak” or “neutral”) [16]. Intention is defined as a conscious decision to perform a behaviour, a resolution to act in a certain way or an impulse for purposeful action [17]. According to the Theory of Planned Behaviour (TPB), a person’s intention is the main determinant of their behaviour [18, 19]. Intention is considered a valid proxy measure for behaviour in the development of implementation interventions [20, 21]. A behaviour change intervention, such as an intervention plan or strategy informed by behavioural theory and evidence, could increase women’s intention to use a PtDA for deciding about prenatal screening.

In health contexts, the TPB is just one of many theories that explain behaviour and so inform methods for developing and evaluating behaviour change interventions [24]. The Theoretical Domains Framework (TDF) was conceived specifically to address these many overlapping behaviour change theories and the lack of guidance as to how to choose between them [17]. It is based on a synthesis of 33 behavioural theories clustered into 14 (originally 12) domains [17, 22]. Starting with behavioural analysis, intervention designers select the domains they wish to investigate in order to design their interventions [22].

Another framework for designing and evaluating interventions is the Behaviour Change Wheel (BCW), based on a synthesis of 19 behaviour change frameworks [23]. The BCW is based on a behaviour model known as COM-B: capacity, opportunity, and motivation, “conditions” considered essential to performing a behaviour [23]. The BCW considers intention (the central component of the TPB) as part of motivation, which it conceptualises broadly to include personal, social and structural motivations and abilities [24]. According to the BCW, changing a behaviour involves using one or more of nine “intervention functions” for addressing a deficiency in one or more of these three conditions [23, 24].

Interventions designed to alter or redirect the causal processes that regulate behaviour may use one or more behaviour change techniques (BCTs). Michie has developed a taxonomy of 93 BCTs [25]. BCTs are increasingly used to design health interventions for a variety of populations and settings [26, 27]. Characterising interventions by their BCTs is helpful in understanding why interventions may be more or less effective, as BCTs are observable, replicable, and irreducible components of the intervention [23]. In designing an intervention, BCTs are chosen to match the specific behaviour change targeted, and the choice should promote the development of more effective and replicable interventions [25]. According to the BCW, relevant functions must be selected, and then specific BCTs chosen to match them. Previous work in areas such as smoking [28] and excessive alcohol consumption [29] shows typically that a combination of BCTs is needed for each targeted behaviour change.

It is currently unclear which BCTs would be relevant for promoting the use of a PtDA in the context of DS prenatal screening. However, the Delanoë study developed a rich database of information about pregnant women’s intentions to use a PtDA for prenatal screening coded to the TDF domains. This database is an important source of evidence about potentially effective BCTs. Michie et al. suggests using the TDF as an option (instead of COM-B) for identifying what needs to change [23]. We hypothesised that if we derived BCTs using the COM-B and the TDF separately, we would find unique BCTs from each process that could improve the intervention.

However, we wanted to base our intervention not only on theory and evidence about effectiveness, but also on the preferences and experiences of the people targeted by our intervention. Inspired by the principle of user-centred design [30], in our second design phase we therefore involved the targeted users.

Thus, the aim of our study was to first identify the most appropriate theory-based BCTs for an intervention to promote the use of a PtDA for DS prenatal screening, and then to explore pregnant women’s perceptions about the acceptability and relevance of these BCTs. We propose that the resulting intervention may improve pregnant women’s intention to adopt these tools for DS screening, and, potentially, their decisional and health outcomes.

## Methods

### Design and study context

We conducted a two-phase qualitative descriptive study: (i) theoretical design phase and (ii) user-centred design phase. Our study was embedded within a larger initiative entitled PEGASUS (PErsonalized Genomics for prenatal Aneuploidy Screening USing maternal blood) designed to enable decision makers, pregnant women and their partners to make informed choices about prenatal genetic screening and diagnosis and to reduce risks associated with amniocentesis [31]. Approval for the current study was obtained from the research ethics boards of the Centre de Santé et de Services Sociaux de la Vieille-Capitale, and the CHU de Québec.

### Theoretical design phase

This phase was guided by the BCW and the behaviour change intervention design process (three stages and eight steps) recommended by Michie [23]. Stage One (Steps 1–4) involves understanding and defining the behaviour; Stage Two (Steps 5–6) consists of identifying the appropriate intervention (defining its functions) and addressing policy categories; and Stage Three (Steps 7–8) consists of identifying appropriate BCTs and mode of delivery. We used the BCW to identify relevant “conditions” (COM-B) and “domains” (TDF) as targets for change (Stage 2); from functions associated with these, we generated a list of appropriate BCTs (Stage 3) [23, 32], to be validated later in the user-centred design phase.

#### Stage 1: Understand the behaviour

##### Steps 1 & 2: Define the problem in behavioural terms and select the target behaviour

Steps 1 & 2 were completed in advance of this study. The behaviour most conducive to shared decision making (SDM) in the context of prenatal screening for DS was identified as PtDA use by pregnant women, referred to hereinafter as “use the PtDA” [16, 33].

##### Step 3: Specify the target intention/behaviour

In the province of Quebec, PtDAs for DS prenatal screening are not routinely used in practice [34]. Furthermore, in the Delanoë study, about one third of the pregnant women offered such a PtDA indicated low intention to use it [16]. The determinants of this intention, according to the TPB, were identified as attitude, anticipated regret, descriptive norm and moral norm [16] (moral norm is how a person evaluates a behaviour in terms of their notions of right and wrong; descriptive norm is how a person evaluates how others would evaluate a behaviour) [18].

##### Step 4: Identify what needs to change

**Fig. 1:**
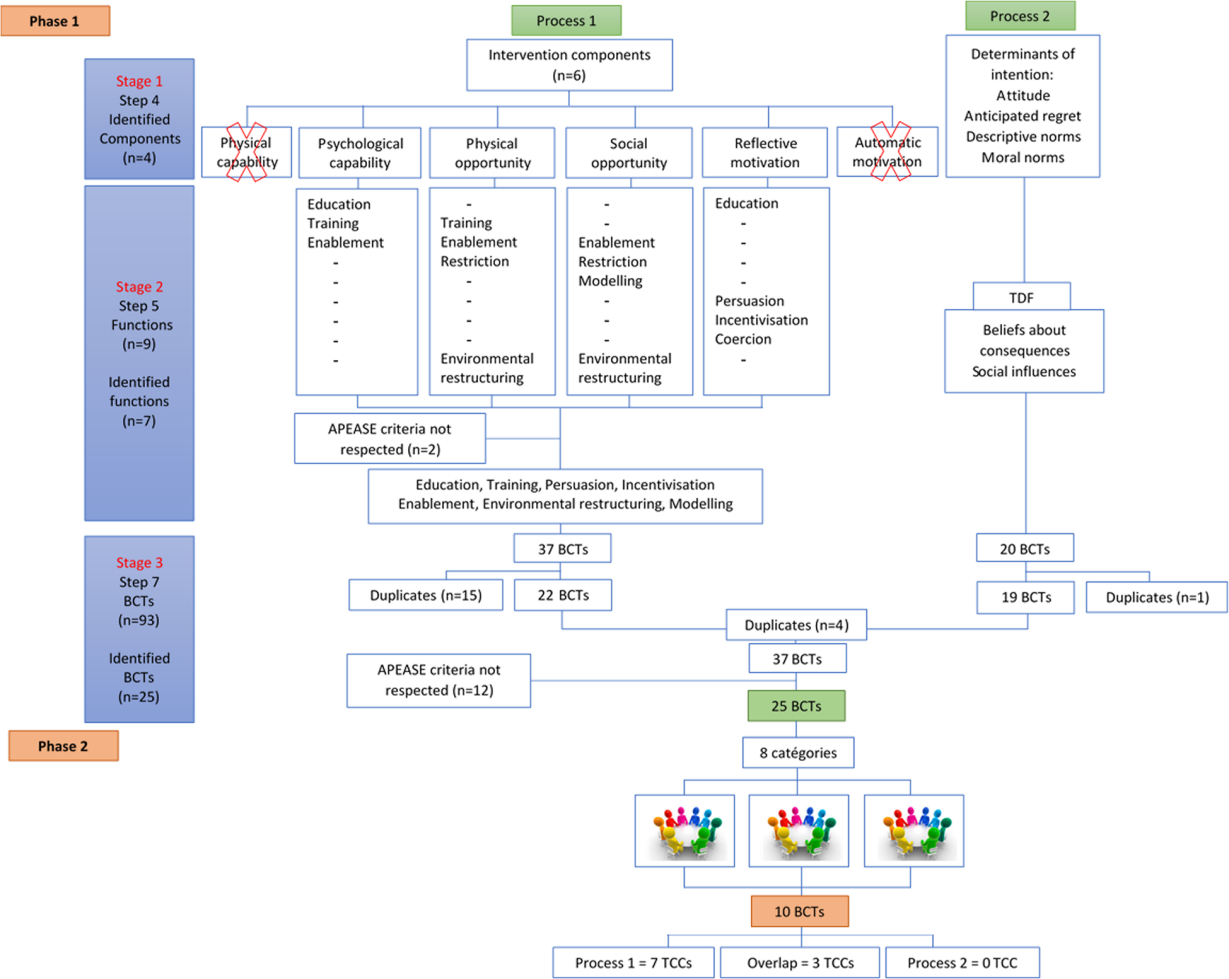
Study phases

We used two processes for this step. The first used the BCW [23, 24], i.e. the COM-B or “conditions” considered necessary for the behaviour to occur (Table 3, Fig. 1). The second used the TDF [23, 24, 35]. In the Delanoë study, the authors used an enriched TPB to identify the four determinants of pregnant women’s intention to use PtDAs [16] (the TPB is one of the 33 theories that contributed to the design of the TDF) [22]. We mapped these four determinants to the “domains” of the TDF to further identify what needed to change.

#### Stage 2: Identify intervention options

##### Step 5: Identify intervention functions

The BCW identifies nine intervention functions, the broad categories of means by which an intervention can change behaviour [23, 24]. We identified those most likely to bring about the needed change in the four COM-B conditions selected. Then, we applied the APEASE criteria (affordability, practicability, effectiveness and cost-effectiveness, acceptability side-effects/safety, equity) as recommended by Michie [23] (Fig. 1).

##### Step 6: Identify policy categories

The BCW also incorporates seven policy categories, i.e. policies that could enable an intervention to occur. We omitted this step because access to political levers was not within the scope of intervention design.

#### Stage 3: Identify content and implementation options

##### Step 7: Identify behavioural change techniques

To ensure we did not miss any important BCTs we used both the TDF and the COM-B model to derive BCTs, thus ensuring the intervention would be informed by two frameworks and a large body of existing evidence (Fig. 1). This would test our hypothesis that both frameworks are necessary and complementary for deriving BCTs.

In Process 1, based on the COM-B model, we selected the most frequently used BCTs associated with our seven selected functions. In Process 2, we identified BCTs related to our two TDF domains. The resulting list of BCTs was discussed by pregnant women in the user-centred design phase (below).

Steps 4, 5 and 7 were performed by five study authors (TTA, SAR, HR, MPG, FL), with combined expertise in medicine, engineering, implementation science, community and public health and shared decision making.

##### Step 8: Identify the mode of delivery

In keeping with user-centred design, we consulted pregnant women about their preferred delivery mode.

### User-centred design phase

In this phase, an extension of Stage Three, we presented the final list of BCTs to a sample of pregnant women to find out which they thought would be acceptable and relevant for using the PtDA and to solicit their suggestions for modes of delivery.

#### Settings, participants and recruitment

In the province of Quebec, about 51% of prenatal care is provided by family physicians, 45% by obstetricians/gynaecologists [36] and 4% by midwives [37]. To ensure a diversity of prenatal care experiences and promote variation in our sampling [38], we purposively recruited pregnant women from a family medicine clinic, a university hospital obstetrics/gynecology department, and a birthing centre in Quebec City, Canada. This strategy allowed us to sample women who had different prenatal care pathways as well as different socio-economic backgrounds (Table 4). We also sought to enhance variation by sampling women based on their parity, i.e. whether they were pregnant for the first time (primiparous) or had given birth to other children (multiparous). Studies suggest that parity may influence prenatal screening decisions [39, 40]. Eligibility criteria included: a) age at least 18 years, b) not less than 16 weeks pregnant or having just given birth, c) having already decided about DS prenatal screening for the current pregnancy, and d) able to speak French. We excluded women who had participated in previous phases of the PEGASUS project related to SDM [16, 33], who presented a high-risk pregnancy (e.g. preeclampsia, gestational diabetes, multiple pregnancy), or whose delivery date was close to the data collection dates. An experienced research assistant and the first author (TTA) met pregnant women in waiting rooms at their prenatal care appointment and invited eligible women to participate in the study.

#### Data collection

We collected data through focus groups because group processes can help women feel comfortable sharing their experiences and views on prenatal care and clarify their individual and collective opinions on relevant BCTs [6–8]. According to Krueger (1994) and Morgan (1997), focus group studies can generally reach saturation after three to six focus groups, although this can vary depending on study objectives and topic complexity [41, 42]. We thus decided to organize three focus groups and then re-evaluate the need for additional groups depending on levels of data and thematic saturation. We aimed to recruit six to eight participants per group [42]. Focus groups were conducted by an experienced moderator (MD) [43] who is a research assistant with an MSc in Public Health accompanied by an assistant moderator (TTA). The moderators had no relationship with participants prior to the study.

To develop our interview guide, we grouped the final list of BCTs into eight categories, based on the BCT taxonomy hierarchy and on similarities between BCTs, for discussion of one category at a time: Information channel, information/content, learning, support, other’s approval, consequences, reward and change (Table 1, Fig. 1). We then developed questions for each category (Table 2) and pilot-tested the guide with a convenience sample of five women.

**Table 1:** BCTs clustered into themes

BCT themes	Number of BCTs	Potential BCTs	Acceptable and relevant BCTs
Information channel	1	Credible source^1^	✓
Information/content	4	Goal setting (behaviour)^1^	✓
		Goal setting (outcome)^1^	✓
		Problem solving^1^	✓
		Action planning^1^	✓
Learning	3	Demonstration of the behaviour^1,2^	✓
		Instruction on how to perform a behaviour^1^	**×**
		Identification of self as role model^2^	**×**
Support	3	Social support (unspecified/general)^1,2^	✓
		Social support (practical)^1,2^	✓
		Social support (emotional)^2^	**×**
Other’s approval	1	Information about others’ approval^2^	**×**
Consequences	9	Information about health consequences^1^	**×**
		Social and environmental consequences^1,2^	**×**
		Emotional consequences^2^	**×**
		Salience of consequences^2^	**×**
		Anticipated regret^2^	**×**
		Comparative imagining of future outcomes^2^	**×**
		Pros and cons^2^	**×**
		Covert sensitisation^2^	**×**
		Covert conditioning^2^	**×**
Reward	1	Social reward^2^	**×**
Change	3	Adding objects to the environment^1^	✓
		Prompts/cues^1^	✓
		Restructuring the physical environment^1^	**×**

^1^ = Process 1; ^2^ = Process 2

**Table 2:** Questions asked of participating pregnant women; identified themes; and representative quotes

Theme	Representative excerpts of quotations	Group in which idea was mentioned	No. of people who mentioned the idea
Theme 1: Information channel - What would be the best way to inform you about the PtDA?			
Desirable information channel			
Healthcare professional	For sure, if the health professional gets it out of the package of material and says this one here, it’s really important that you fill it in. I think I’d maybe pay a bit more attention, I’d begin with that document first. In fact I think that’s the only way I’d read the whole thing through (FG2, participant 6).	3	12
Internet: Official websites	I’d expect to find that kind of information on a government health website (FG1, participant 1).	3	8
Healthcare professional + Internet	You can reach people on the Internet. Information is more accessible there. But there are people who won’t use the Internet to get that information. So for those people, who maybe don’t know how to try and get the tool, or don’t want to and don’t see the need to get the information, if it’s the doctor who gives it to them, well then… (FG1, participant 2)	2	4
Video on Youtube	But if not maybe a video on Youtube which the doctor could refer you to, [a web address] in the documents at the end. If you want more information, in fact, you could go see the tutorials on Youtube, but otherwise there isn’t anywhere else (FG2, participant 4)	2	2
Undesirable information channel			
Social networks	I hate to say, but [don’t use social networks] so you don’t lose credibility. You know, if it’s on the social networks you’d lose some (FG2, participant 7).	2	4
The Internet	I won’t go looking for it [on the internet]. I’d wait for someone to give it to me, because it’s not up to me to go looking for that stuff. You’ve made time in your schedule for your appointments so they should be the ones to give you the information and to know they have to give you this material (FG2, participant 9).	1	3
Theme 2: Information/content - What information/explanations would you like to get to help you use the PtDA?			
Helpful information			
Add PtDA to documents	But especially now where we already have so much information on paper, so that these documents are our source of information, but to only have the information somewhere else, it’s not very helpful, to have to go find the information on another platform so that … (FG2, participant 6).	1	3
Goal of the PtDA	Perhaps just add what the tool is for. Like, this tool will help you make an informed choice, it’s for making a decision that you’re comfortable with. See, just highlight what the tool is for, what you can do with it, without necessarily saying watch out if you don’t you it (laughs)(FG1, participant 2).	1	2
Resources available	Well maybe say that if you read this and have some questions, here’s where you can go… what are the resources if you have questions, if you still don’t know after reading it …(FG3, participant 14).	1	2
Information about availability	If he doesn’t tell me about it, or give it to me, but if he tells me where I can get it, yes (FG3, participant 2).	1	1
Follow-up would be effective	I think that would give the tool more weight. The doctor does a follow-up, and can check that the person has understood … it’s an important decision to make and I’m supporting you and respect the choices you make. I think that makes sense, to give the documents and then [there’s a follow-up]… (FG3, participant 13).	2	3
Closeness of consultations	I’m not sure where this is going, but say you see the doctor once, then the second time you see him, he does the prescribing, that makes sense. But the trouble is, you don’t get two appointments really close together so that may not work (FG3, participant 1).	2	2
Unhelpful information			
Time to read PtDA	No, you know personally I would take the time but perhaps I don’t represent the majority (laughs). I personally would have taken the time to read the whole thing, though. It’s very visual (FG3, participant 15).	2	4
Theme 3: Learning - Would it be relevant to demonstrate how to use the PtDA?			
Demonstration is unnecessary			
A demonstration is not necessary	Not for me, no, this is enough, honestly, I don’t see the point of a video (FG3, participant 15).	8	8
Demonstration could be necessary			
Demonstration in some cases	Exactly, for someone who’s overwhelmed by their pregnancy, perhaps being pregnant took a lot of getting used to, someone who has a condition, perhaps it would help them to have extra support other than just being told to read it. I’m trying to think, it wouldn’t be bad but I think presenting [the video] systematically every time, that’s not a great idea (FG3, participant 13).	2	2
Healthcare professional to judge the need for demonstration	I think it’s mostly the health professional who should judge whether the person needs it or not (FG3, participant 14).	1	1
Theme 4: Support - Do you think you would use the PtDA by yourself or with the help of someone else?			
Support is needed			
Decision as a couple	Because we decided together about having a child (laughs) and to be responsible for it, and if it’s a family decision, then it’s for both of us to make … and from my point of view, it’s to involve [the other person] as much [as possible] … if the test is positive, then there are all the consequences that go with that choice. They’ve got to be connected (FG3, participant 13).	3	7
Read with partner or relative	It’s the same for me. Having it in my hands and being able to discuss it somewhere other than in the doctor’s office, even if in our case, we’ve already thought about it, but sometimes it brings up other possibilities, it really shows the way to go and it’s good to have it and to be able to discuss it with my partner (FG3, participant 13).	2	6
In case of misunderstandings	I would use it with my partner too. Look, if there are little things we don’t understand, after thinking about it, and if we had questions, we’d go and see the doctor again to understand it better, and then make our decision (FG1, participant 1).	1	2
Support is not needed			
Read alone	No I think it’s maybe better to read it by yourself without someone commenting every 30 s on what you’re reading. Because that’ll influence you. Say you’re reading it together and for example he says, well, maybe not, you get comments and it can kind of influence your judgement (FG1, participant 2).	2	6
Theme 5: Other’s approval - What do you think of the opinions of other people on the use of the PtDA?			
Other’s approval (pregnant women, relatives…)	Not for me personally, no, because someone might be pregnant in your group, or even let’s say a mother, it’s more to reassure you; you make friends and yes you talk all the time but I’d never just rely on a mother for that; anyway, we’re all different, even the things I say I’m not the same as others … Having the approval of other people is worth nothing. It’s worth absolutely zero (FG2, participant 7).	3	6
Healthcare professional’s approval is implied	For sure you have to go through that stage. Then when you get there he automatically gives it to you. My doctor gave it to me so I imagine he thinks it’s worth something (FG3, participant 15).	1	1
Thème 6: Consequences - What do you think the consequences would be (advantages, disadvantages) of using the PtDA, or of not using it? Would you like more information about the advantages/disadvantages of using the PtDA or not using it?			
Consequences (advantages and disadvantages) of using the PtDA			
Advantages of using the PtDA	Yes, there’s enough information on it. But to have it, and to know the statistics, I think that’s great, I have to say. The advantages and disadvantages too, you know what you’re up against, look it’s really clear, and that’s really helpful (FG3, participant 15).	3	13
Disadvantages of using the PtDA	The doctor tells you want to do, like she just said, and you think you’ll just do the test but you don’t know anything about what that means. But then when you know everything that could happen, it makes you even more worried (FG1, participant 3).	3	8
Information about consequences			
Information about consequences is not necessary	Look, people get into a panic when you get too negative … (FG1, participant 5).	2	7
Theme 7: Reward - Do you think a reward is needed for using the PtDA?			
No need for a reward	No, I see it as a tool that is there to help you, so I don’t see why you’d need a reward for using it. I mean, if people don’t want to use it, then they don’t have to! (laughs) But I see it as an aid, so I think if you take it, it’s because you want the information, not because you feel obliged to use it (FG1, participant 2).	3	11
A reward wouldn’t change anything	For me what’s important about this tool is that you use it and then it makes you think about things, and know more [what you want]. From the moment I read it and did it, personally it gave me something. Even if my doctor was really pleased I did it, that wouldn’t make much difference, it wouldn’t add much. It did its job when I took the time to fill it out (FG3, participant 13).	3	2
Healthcare professional could offer positive reinforcement	Yes okay the doctor could do that, he could go, I’d really like that [if you] used a tool I suggested, but they do positive reinforcement without realizing it, they might say it’s great that you used it, it’s something they’d say without even thinking about it, I don’t know if that’s what you mean by a reward, but it often happens that just saying it’s good you used it … (FG1, participant 5)	2	2
The PtDA is its own reward	The reward is having the tools and having the information. We don’t need a trophy! (laughs), we don’t need a reward you know (FG1, participant 4).	2	2
Theme 8: Change - What kind of changes could be made in the places you go to encourage you to use the PtDA?			
Suggested changes for the use of the PtDA			
Screen	Screens work really well, better than posters (FG3, participant 14).	2	2
Poster	And like we were just saying, perhaps a poster in public places where pregnant people are likely to go, at least a poster that could at least tell you there’s this information you can get. And where it’s available. That’s the thing. (FG1, participant 1).	2	2
Places to make changes			
Medical environment	I don’t really see it outside a medical setting either (FG3, participant 15).	2	3
In the hospital	Yes I think a sign in the waiting room that summarizes what’s inside it, not like an ad, but as information, that could be good because you’re sitting waiting in the waiting room and I read what’s going across the screen, but not an ad, really a summary of what’s inside it, in the information (FG1, participant 4).	2	3

At the beginning of each focus group, we showed a 10-min video showing the use of the PtDA by a pregnant woman and her partner in a simulated prenatal follow-up encounter with a healthcare professional. The moderators also gave PtDAs to participants. Discussions were recorded with participants’ consent, and audio recordings transcribed verbatim. The assistant moderator took notes on any additional relevant remarks during each encounter. We planned 60 to 90 min for each group. At the end each participant completed an individual socio-demographics questionnaire (Table 4).

#### Data analyses

Four authors (ATT, MM, MD and SAR) performed deductive and inductive thematic analyses of verbatim transcripts [44] following a step-by-step guide [45]. They independently read through the transcripts to familiarise themselves with the data and attached initial codes according to the most basic elements of the raw data. Authors then met to analyse and cross-check their initial codes, which were matched to BCT categories when possible. Some codes fit easily into the BCT categories, others did not. Discrepancies were discussed and resolved during three team meetings. We validated the resulting BCTs in each category with the original list of BCTs. All analyses were conducted in French and then relevant quotations illustrating each category were extracted into a summary table, which was translated into English by a professional translator (Table 2) [46].

## Results

We report here findings from the focus groups that have a bearing on intervention development. Similar items have been grouped together.

### Phase 1- theoretical design phase

#### Stage 1: Understand the behaviour

As noted above, Steps 1 to 3 were undertaken before the beginning of the study. The target behaviour was use of a PtDA about DS screening by pregnant women and their partners (if indicated) during prenatal follow up [16, 33].

##### Step 4: Identify what needs to change

From the COM-B, we selected “psychological capability” (knows how to use it), “physical opportunity” (it is available), “social opportunity” (can see others using it) and “reflective motivation” (believes it could be useful) (see Table 3, Fig. 1). We excluded “physical capacity”, assuming that most women would have no physical limitations to using the PtDA. We excluded “automatic motivation” (has a habit of using it) because using the PtDA is essentially a one-time behaviour in this context. With the TDF process we identified the domains “beliefs about consequences” (attitude and anticipated regret); and “social influences” (moral and descriptive norms) [17, 22, 35]. Thus we ended up with four “conditions” from the COM-B model and two “domains” from the TDF.

**Table 3:** Use of the model COM-B to identify the components of the intervention

	Components COM-B	What needs to happen for the target behaviour to occur?	Is there a need for change?
CAPABILITY (C)	Physical capability	Have the physical capacity to read the PtDA.	No. The reading of the PtDA doesn’t require special physical skills.
	Psychological capability	Know how to use the PtDA.	Yes. In another study, more than half of the participants did not did not know of any PtDAs, therefore did not know how they are used.
OPPORTUNITY (O)	Physical opportunity	The PtDA is available.	Yes. For the tool to be used, it must be available.
	Social opportunity	Have the opportunity to see other women use the PtDA.	Yes. As the PtDA is not available yet, pregnant women have not heard about it or had the opportunity to see anyone else using it.
MOTIVATION (M)	Reflective motivation	Believes that using the PtDA could be useful.	Yes. In another study, more than half of the participants did not know of any PtDAs, therefore did not know it could be useful.
	Automatic motivation	Have established a routine and a habit to use the PtDA.	No. It is a one-time behaviour so there is no need for it to become habitual.
	Diagnosis of COM-B components	Psychological capability, physical opportunity, social opportunity and reflective motivation are components needed in an intervention to increase women’s intention to use the PtDA.	

#### Stage 2: Identify intervention options

##### Step 5: Identify intervention functions

We retained seven of the nine functions: education, training, persuasion, incentivisation, enablement, environmental restructuring and modelling (Fig. 1). We excluded coercion and restriction because these presented ethical issues in this context.

##### Step 6: Identify policy categories

As indicated above, we did not complete this step. However, political categories relevant to scaling up such an intervention could be communication and marketing, practical guides, regulation, environmental and social planning and provision of service.

#### Stage 3: Identify content and implementation options

##### Step 7: Identify behavioural change techniques

In Process 1, based on the COM-B model, we selected the BCTs most frequently associated with our seven functions. Out of 37 BCTs, we retained 22 after removing duplicates. In Process 2, we chose BCTs that matched our two TDF domains. Of the 20 BCTs resulting from Process 2, 19 were left after removing duplicates. A total of 41 BCTs therefore resulted from these combined processes. After applying the APEASE criteria and removing inter-process duplicates, we had a list of 25 BCTs (Fig. 1, Table 1): 10 from Process 1 and 11 from Process 2, with four BCTs overlapping both processes.

### Phase 2 – User-centred design phase

#### Participant characteristics

We recruited women between February and March 2017. We approached a total of 56 women, of whom 18 refused to participate because they were not interested in the topic or not available for the focus groups. Two women were not eligible because of high-risk pregnancy. Thirty-six women agreed to participate but 16 were unable to participate in a focus group on any of the dates proposed and so instead were invited to participate in a later phase of the study. Five women were expected to participate but withdrew on the days of focus groups for personal or health reasons, leaving a total of 15 women in the three focus groups. The average meeting length was 68 min. Five women participated in the first focus group (all primiparous), seven in the second (five primiparous and two multiparous) and three women in the third (all multiparous). Their mean age was 27 years (range 19–43 years) and their pregnancy term was 19–36 weeks. One woman had just given birth. Of the 15 participants, one had decided to not undergo DS prenatal testing and three reported difficulties in making the decision (Table 4).

**Table 4:** Participant characteristics (*n* = 15)

Characteristics	*n* (%)
Age, years	
Mean	26.8
Range	19–43
Term pregnancy (weeks)	19–36
Ethnicity	
White	12 (80)
African	2 (13)
Latin American	1 (7)
Education	
No high school	1 (7)
High school diploma	2 (13)
Collegial diploma	6 (40)
University degree	6 (40)
Parity	
Primiparous	10 (67)
Multiparous	5 (34)
Screening for Down syndrome	
Yes	14 (93)
No	1 (7)
Felt it was difficult to make decision	
Yes	3 (20)
No	12 (80)
Decisional support	
Partner	11 (73)
Friend/family	1 (7)
Alone	3 (20)
Civil status	
Single	2 (13)
Not single	13 (87)
Annual family income	
< $18,000	1 (7)
$18,000 - $29,999	3 (20)
$30,000 - $59,999	6 (40)
> $60,000	4 (26)
No answer	1 (7)

#### Principal findings

We present results organised by BCT category, followed by additional comments. Quotations illustrating each category are reported in Table 2.

##### Category 1: Information channel

Focus groups agreed they would prefer to receive the PtDA from their healthcare professional during their first prenatal visit. While all types of health professional were seen as reliable information sources, participants said visits with physicians were shorter than those with midwives and that physicians did not always properly explain the pros and cons of DS prenatal screening. Many participants routinely turned to the internet to find information about their pregnancy, though did not always find it easy to find reliable information online. A number of participants said they would not look for the PtDA on the internet. Others considered official hospital or government websites as the second most trustworthy source of information, after health professionals. Even though all participants said they consulted a health professional for prenatal care, they suggested that information both on the internet and from health professionals would reach more women and give them more choice. A YouTube video recommended by the health professional could combine both (Table 2).

##### Category 2: Information/content

Participants felt health professionals should clearly explain the goal of the PtDA when asking them to use it. They also wanted to know who to talk to if they had questions after reading it. If they didn’t receive the PtDA from the health professional, they wanted to know how to access it (e.g. given a link to a website). Participants suggested that the PtDA be included in the information package they receive during the first consultation. They did not see the need to state how long it would take to read the PtDA. Several participants said they typically read all the documents given them, however long, because they wanted to be as informed as possible about their pregnancy and their child’s health.

Many participants suggested that after receiving the PtDA, they would need a follow-up discussion with their health professional before making a decision. This was considered a strong motivator to reading the PtDA and would give the tool importance (Table 2).

##### Category 3: Learning

None of our participants felt they needed to be taught how to use the PtDA, or needed a demonstration. However, they said a demonstration would be useful if women had questions or particular needs (e.g. low literacy) (Table 2).

##### Category 4: Social support

While some participants stated they would use the PtDA with their partner, others said they would use it alone. However, all agreed that the decision to undergo DS screening involves both the pregnant woman and her partner. While participants expressed a desire for input and decisional support from their partners or family members (if they were single), they also felt they were the ones ultimately responsible for making final decisions about screening and their baby’s health. Participants also said they would need support from their health professional if they had questions after using the PtDA (Table 2).

##### Category 5: Approval of others

Participants reported that health professionals’ approval about using the PtDA was important but implicit. They felt that the approval of other people (such as other pregnant women, friends or parents) would not affect their decision, and that they could judge by themselves if the PtDA would be helpful (Table 2).

##### Category 6: Consequences

Participants perceived the advantages of using the PtDA as being that it could help them to make a better decision, save time (rather than searching for information online), understand the risks of screening better, and help them prepare for the next steps in the screening process. In terms of disadvantages, a number of participants said using the PtDA could cause anxiety or make them feel emotionally fragile, lengthen the consultation time, and increase paper clutter. However, they thought it unnecessary to include information about consequences, especially negative consequences, in an intervention. They felt that this would make women anxious, or pressured to read the PtDA. Simply providing information about the goals of the PtDA was preferable (Table 2).

##### Category 7: Reward

All participants agreed that a reward would not convince women to use the PtDA. The PtDA was perceived as a reward in itself as it would save women time in gathering information about DS prenatal testing. Being congratulated by their health professional for using the PtDA was not a motivating factor, either (Table 2).

##### Category 8: Environmental change

Participants suggested that changes could be made in hospital waiting rooms because pregnant women spend a lot of time there. They suggested a message on the TV screen and/or a poster advertisement for the PtDA. Almost all participants added that if these messages were elsewhere than in hospitals or pharmacies they would not attract their attention (Table 2).

##### Additional comments

The participants made other comments that did not fit into the categories above but which appeared relevant to the development of the intervention. They spoke about knowing nothing about PtDAs prior to the study, their previous and current experience of pregnancy, and the lack of SDM in prenatal services in family practice and gynecology. They suggested the need to train health professionals in engaging patients in SDM about DS prenatal screening.

#### Summary of retained BCTs and functions

The theoretical phase resulted in 25 BCTs, and the participants perceived the acceptability and relevance of 10 of these: 1) using credible sources, 2) setting goals around the targeted behaviour, 3) setting goals around the outcomes of the behaviour, 4) encouraging problem solving around barriers/facilitators to adopting the behaviour, 5) action planning for the target behaviour, 6) ensuring general/decisional social support, 7) ensuring practical social support, 8) demonstrating the target behaviour, 9) providing prompts/cues, and 10) adding objects to the environment in which the behaviour should take place (Fig. 1, Table 1).

All 10 BCTs judged acceptable and relevant by the participants had been identified through Process 1, based on the COM-B. In Process 2, based on the TDF, none of the BCTs related to the domain “beliefs about consequences” were judged acceptable or relevant. Among the 10 BCTs related to the domain “social influences”, eight met the APEASE criteria and three were judged acceptable and relevant by the women. These three BCTs had also been identified through Process 1, i.e. would have been identified without Process 2. The 10 retained BCTs were related to the BCW functions of education, training, enablement, persuasion, modelling and environmental restructuring. Of the seven BCW functions, incentivisation was the only one the participants considered unlikely to be effective for a future intervention.

## Discussion

This theory-informed qualitative study aimed to fine-tune a method for identifying BCTs to be incorporated into an intervention to promote use of a PtDA for DS prenatal screening. We used two theory-based frameworks to derive relevant BCTs and added a user-centre design dimension to identify which of these BCTs the pregnant women found acceptable and relevant. This approach led us to focus on 10 BCTs. Our results lead us to make five observations.

First, regarding our method, we had hypothesised that BCTs derived from the TDF would be necessary for our intervention, but we found that the BCTs derived directly from the TDF process were replicated through the BCW process. This implies that the BCW may be comprehensive enough to be able to dispense with the TDF option (Step 4) suggested in the BCW method. Also, we borrowed methods from user-centred design and involved pregnant women, i.e. end-users of the intervention, to select appropriate BCTs [47]. An intervention focusing on their needs and preferences is likely to be more acceptable, feasible and effective [47]. In fact, they had the final say in defining the content of the intervention. Even if it takes more time, as discussed below, the women’s contribution was innovative and relevant for informing intervention design.

Second, participating women found that 10 theory-derived BCTs were acceptable and relevant for use in an intervention to promote the use of a PtDA by women facing prenatal screening decisions. A systematic review of changing physical activity behaviour suggested that an intervention with 10 or more BCTs is more likely to succeed than one with fewer than 10 [48]. Some of our BCTs, such as “goals and planning” [49, 50] and “social support” [51], have been included in interventions targeting pregnant women for other behaviours. In fact, “goals and planning” is one of the most commonly used BCTs and one of the most successful for diet interventions (gestational weight management) and mixed interventions [49]. However, the BCT “social reward”, found in a list of BCTs for smoking cessation [28] and for reduction of excessive alcohol consumption [29], is not on our list. Women proposed decisional support as a BCT, which we classified under the BCT “general social support”. Clearly, an intervention in the context of DS prenatal screening needs a precise combination of BCTs. Our findings could be helpful for establishing a taxonomy of BCTs likely to be effective specifically for SDM interventions.

Third, participants proposed that a motivating factor for all pregnant women would be planned follow-up, which fits with the BCT “action planning”. This is different from recent studies on screening decisions which propose strategies tailored to each individual to address motivations, confidence, and barriers [8]. Women in our study did not see the need for tailored strategies, except for demonstrating the PtDA for women with special needs. This confirms results of other studies showing that pregnant women with lower health literacy levels may need more tailored interventions to increase their likelihood of using a PtDA [16, 18]. The fact that participants thought tailoring unnecessary would make the intervention easier to operationalise, as it could be more generic.

Fourth, regarding intention, BCTs selected by the women in our study, “problem solving” (which includes “coping planning” [25]) and “action planning”, are strategies usually used to support implementation, i.e. to move from intention to behaviour [52–54]. Originally, we aimed to increase the level of intention of women with little intention to use a PtDA for prenatal screening (31.5% in the Delanoë study). But an intervention based on the BCTs suggested by our participants could serve to design an intervention suited for all pregnant women, whatever their level of intention [16]. While intention is often used as a proxy for behaviour, the women’s selection of these BCTs is in keeping with criticisms of behavioural theories based on intention as proxy increasingly found in the literature [52].

Lastly, our final list only included 10 out of the 25 original BCTs, reducing the complexity of a potential intervention. However, this does not imply relevant BCW functions were left out (they selected six out of seven). The BCTs identified covered a range of intervention functions, ultimately illustrating the possible need for a multi-component intervention that targets several determinants of behaviour change. This would create a more convincing intervention than is typically observed in other SDM interventions in which a PtDA is used, which focus mostly on the functions of education and training [55–58]. This was the case, for example, in a study aiming to improve SDM for women deciding about prenatal testing for foetal abnormalities [59], in which a health professional gave women the PtDA but no additional decision support and no follow up [59]. Indeed, distribution of educational materials alone is one of the most common strategies found across these studies [60]. In contrast, in our study, many of the BCTs retained by the women necessarily involved the healthcare professional, for example, an action plan, and practical social support. This suggests that the most appropriate intervention would be an intervention plan in the clinical pathway of pregnant women that is implemented by the healthcare professional. The next step will require investigating the various clinical pathways of pregnant women so that the plan can be adapted to each type of prenatal service.

### Limitations

Our study has a few limitations. First, only three women participated in one of the focus groups, lower than the minimum of four participants recommended by some authors [61]. This stemmed from difficulties recruiting and securing the participation of multiparous women, who were less available because of family responsibilities or appointments. While we made efforts to accommodate these women (flexible dates, compensation for travel and parking), we should have anticipated higher withdrawal rates given the demanding schedules of these women. With more multiparous women in our sample, we would be more confident that their choice of BCTs aligned with that of primiparous women. Second, we diversified our sample by recruiting women with different care providers, socioeconomic status, ages and parity. However, some populations are not well represented in our sample, notably those with lower education and those who declined DS screening. Finally, for ethical reasons and out of respect for patient autonomy we eliminated BCTs related to restriction and coercion before women could consider their relevance. In fact, women’s access to PtDAs can be restricted (e.g. by health professionals), or they could be obliged to use them, so future studies could consider women’s views on these particular BCTs.

## Conclusion

This is the first study validating the derivation of BCTs using the BCW (with the TDF option) for an intervention to promote the use of a PtDA in the context of DS prenatal screening, and which includes pregnant women’s views on their acceptability and relevance. The TDF option proved unnecessary. Pregnant women considered 10 BCTs acceptable and relevant. A future intervention will be suitable for pregnant women whatever their level of intention to use the PtDA. Further work is needed to adapt the intervention to each prenatal care clinical pathway.

## Abbreviations

APEASE: Affordability, practicability, effectiveness and cost-effectiveness, acceptability side-effects/safety, equity
BCT: Behaviour change techniques
BCW: Behaviour change wheel
DS: Down syndrome
PEGASUS: Personalised Genomics for Prenatal Aneuploidy Screening Using Maternal Blood
PtDA: Patient decision aid
SDM: Shared decision making
TDF: Theoretical domains framework
